# Complete plasmid sequence carrying type IV-like and type VII secretion systems from an atypical mycobacteria strain

**DOI:** 10.1590/0074-02760160546

**Published:** 2017-07

**Authors:** Sergio Mascarenhas Morgado, Michel Abanto Marín, Fernanda S Freitas, Erica Lourenço Fonseca, Ana Carolina Paulo Vicente

**Affiliations:** Fundação Oswaldo Cruz-Fiocruz, Instituto Oswaldo Cruz, Laboratório de Genética Molecular de Microrganismos, Rio de Janeiro, RJ, Brasil

**Keywords:** fast-growing mycobacteria plasmid, secretion system, conjugation

## Abstract

The genus *Mycobacterium* is highly diverse and ubiquitous in nature, comprehending fast- and slow-growing species with distinct impact in public health. The plasmid-mediated horizontal gene transfer represents one of the major events in bacteria evolution. Here, we report the complete sequence of a 160,489 bp circular plasmid (pCBMA213_2) from an atypical and fast-growing environmental mycobacteria. This is a unique plasmid, in comparison with the characterised mycobacteria plasmids, harboring a type IV-like and ESX-P2 type VII secretion systems. pCBMA213_2 can be further explored for evolutionary and conjugation studies as well as a tool to manipulate DNA within this bacteria genus.

Plasmids are genetic elements that make up bacteria mobilome involved in horizontal gene transfer (HGT) events. Conjugative plasmids are characterised by the presence of the mobility (MOB) genes region, defined by the origin of replication (oriT) and a relaxase gene, and the type IV secretion system (T4SS)-like genes (Smillie et al. 2010).

The *Mycobacterium* genus encompasses more than a hundred species including fast- and slow-growing organisms, and the latter contains the majority of opportunistic and human pathogens. However, only few plasmids have been described in mycobacteria, and conjugative elements have been rarely reported (Ummels et al. 2014, Uchiya et al. 2015). In fact, conjugation in mycobacteria is just being elucidated (Gray et al. 2016). Interestingly, some mycobacteria plasmids carry genes resembling the type VII secretion system (T7SS) or ESX-P, which is the unique secretion system recognised in *Mycobacterium* genus. In *Mycobacterium tuberculosis* five ESX types (ESX-1-5) were identified based on the ESX core components (EccB, EccC, EccD, and MycP) and on particular variations in the gene content and organisation (Costa et al. 2015, Simeone et al. 2015). The ESX types evolved by gene duplication, and plasmids have been proposed to be a key factor in their radiation into this genus (Dumas et al. 2016, Newton-Foot et al. 2016).

In this study, we determined the complete sequence of pCBMA213_2 plasmid (accession no. KY349138) carried by an environmental fast-growing Mycobacteria strain. The *Mycobacterium sp*. CBMA213 was isolated from the Atlantic Forest soil and characterised by multilocus sequence analysis (MLSA) as belonging to a new species of *Mycobacterium* (data not shown). The pCBMA213_2 sequence was obtained in the context of the CBMA213 whole genome sequencing, using Nextera paired-end library on Illumina Hiseq 2500. Reads were filtered and correted using NGSQCToolkit v.2.3.3 (Patel & Jain 2012) and Quake v.0.3 (Kelley et al. 2010). *De novo* assembly was done using SPAdes v.3.9 (Bankevich et al. 2012). A total of 57 contigs were obtained, and among them, a 160 kb-contig was putatively characterised as a plasmid by the presence of mycobacteria plasmid gene markers, such as relaxase and *rep*A (Ummels et al. 2014, Uchiya et al. 2015). Moreover, its circular topology defined by paired-end information characterised this contig as a replicon. This plasmid was mapped and reassembled with a mean coverage of 144x. A final step of correction was performed using Pilon v.1.20 (Walker et al. 2014). A Maximum Likelihood phylogeny was constructed based on concatenated EccA-E and MycP amino acid sequences (4,700 aa) using Seaview v.4.6.1 (Gouy et al. 2010).

The pCBMA213_2 is a circular plasmid of 160,489 bp, with a guanine-cytosine content (GC content) of 65.9%, characteristic of *Mycobacterium* genus and similar to GC content (65.4%) of CBMA213 genome. The annotation using Prokka v.1.11 (Seemann 2014) identified 161 coding DNA sequence (CDS), and BLASTn analyses revealed that pCBMA213_2 was unique relative to other mycobacterial plasmids considering the overall gene content. However, genes sharing identity with elements related with plasmid conjugation (*vir*D4, *vir*B4, *tcp*C) and mobilisation (relaxase), as well as a set of genes resembling an entire T7SS, were identified among these 161 CDS (Fig. 1). The pCBMA213_2 T7SS genes share the same synteny and 68% identity with the *Mycobacterium sp.* KMS pMKMS01. Indeed, a phylogenetic reconstruction based on EccA-E and MycP proteins, recovered from mycobacteria NCBI database, reveled that pCBMA213_2 belongs to a cluster (ESX-P2), with three other mycobacteria plasmids, and this cluster is related to *M. tuberculosis* chromosomal ESX-2 system (Fig. 2).

**Fig. 1:**
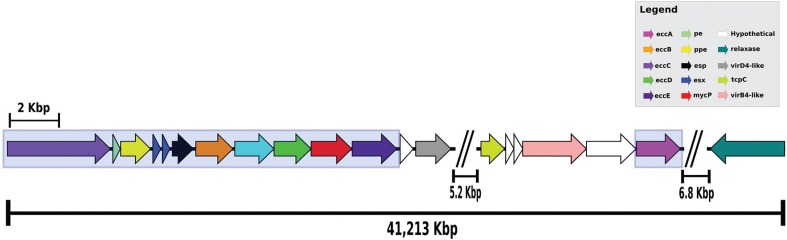
: gene organisation of T7SS, T4S-like and relaxase *loci*, separated by segments with hypothetical genes, in pCBMA213_2. T7SS genes are depicted by box. This segment contains 41,213 kb.

**Fig. 2:**
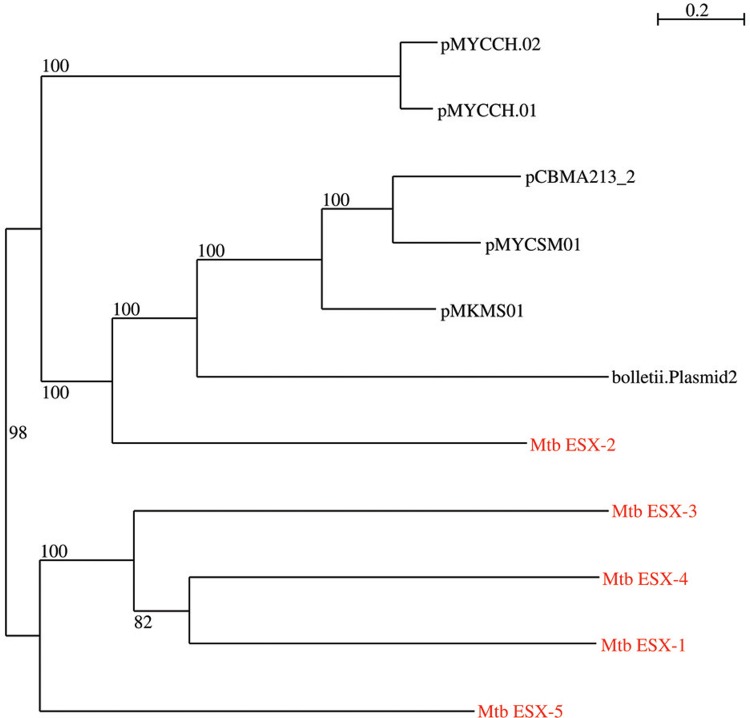
: maximum likelihood phylogenetic tree of T7SS *loci* from mycobacteria plasmids and *M. tuberculosis* (Mtb), based on concatenated EccA-E and MycP amino acid sequences (100 bootstrap replications). Chromosomal ESX 1-5 is colored in red.

In relation to T4S conjugation system, homologues of *tra*I relaxase (*pCBMA213_2_00112*), *vir*B4 (*pCBMA213_2_00101*), *vir*D4 (*pCBMA213_2_00091*) and *tcp*C (*pCBMA213_2_00098*) genes are encoded in pCBMA213_2, but some other T4SS components were not identified. The genes from both secretion systems are distributed in a 41,213 kb segment. Interestingly, Ummels et al. (2014) characterised a novel functional conjugative plasmid in a *M. marinum* that combines elements from both type IV and type VII secretion systems enabling its transfer only between slow-growing mycobacteria. The pCBMA213_2, harbored by a fast-growing mycobacteria, contains this same set of elements, indicating that this plasmid belongs to this new class of conjugative plasmid.

Noteworthy, this plasmid carries the *whi*B6 (*pCBMA213_2_00051*) that has been recently demonstrated to be involved in the regulation of ESAT-6 production and secretion. ESAT-6 is the virulence factor secreted by ESX-1 secretion system in *M. tuberculosis* (Solans et al. 2014, Chen et al. 2016).

Considering the mycobacteria plasmids available at GenBank, pCBMA213_2 presented the highest identity and coverage with pMYCSM01 from a fast growing *M. smegmatis* strain isolated from a human soft tissue lesion (Fig. 2). They share high identity within T7SS *loci* subtilisin gene and three hypothetical protein genes.

Therefore, pCBMA213_2 can be further explored as a model for conjugation studies and/or a tool to introduce DNA in fast-growing mycobacteria.
